# 6-(4-Chloro­phen­yl)-2-isobutyl­imidazo[2,1-*b*][1,3,4]thia­diazole

**DOI:** 10.1107/S1600536810052621

**Published:** 2010-12-24

**Authors:** Hoong-Kun Fun, Madhukar Hemamalini, D. Jagadeesh Prasad, G. K. Nagaraja, V. V. Anitha

**Affiliations:** a X-ray Crystallography Unit, School of Physics, Universiti Sains Malaysia, 11800 USM, Penang, Malaysia; b Department of Chemistry, Mangalore University, Mangalore, Karnataka, India

## Abstract

In the title compound, C_14_H_14_ClN_3_S, the imidazo[2,1-*b*][1,3,4]thia­diazole system is essentially planar, with a maximum deviation of 0.006 (2) Å. The dihedral angle between the imidazo[2,1-*b*][1,3,4]thia­diazole and chloro­phenyl rings is 5.07 (8)°. In the crystal, there are no classical hydrogen bonds but stabilization is provided by weak π–π [centroid–centroid distance = 3.5697 (11) Å] and C—H⋯π inter­actions.

## Related literature

For applications of imidazo [2,1-b]-1,3,4-thia­diazole derivatives, see: Terzioglu & Gursoy (2003 ▶); Kolavi *et al.* (2006 ▶); Gadad *et al.* (2000 ▶); Andotra *et al.* (1997 ▶); Khazi *et al.* (1996 ▶); Andreani *et al.* (1982 ▶,1987 ▶,1991 ▶); Eberle & Robert (1977 ▶).
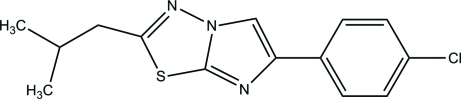


## Experimental

### 

#### Crystal data

C_14_H_14_ClN_3_S
*M* *_r_* = 291.79
Monoclinic, 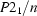

*a* = 5.7552 (1) Å
*b* = 26.4052 (5) Å
*c* = 9.7662 (2) Å
β = 101.388 (1)°
*V* = 1454.92 (5) Å^3^
*Z* = 4
Mo *K*α radiation
μ = 0.40 mm^−1^
*T* = 296 K
0.47 × 0.31 × 0.28 mm

#### Data collection

Bruker SMART APEXII CCD area-detector diffractometer
Absorption correction: multi-scan (*SADABS*; Bruker, 2009) ▶ *T*_min_ = 0.835, *T*_max_ = 0.897
12320 measured reflections
3353 independent reflections
2646 reflections with *I* > 2σ(*I*)
*R*_int_ = 0.023

#### Refinement

*R*[*F* ^2^ > 2σ(*F* ^2^)] = 0.050
*wR*(*F* ^2^) = 0.135
*S* = 1.05
3353 reflections
172 parameters
H-atom parameters constrained
Δρ_max_ = 0.23 e Å^−3^
Δρ_min_ = −0.32 e Å^−3^

### 

Data collection: *APEX2* (Bruker, 2009 ▶); cell refinement: *SAINT* (Bruker, 2009 ▶); data reduction: *SAINT*; program(s) used to solve structure: *SHELXTL* (Sheldrick, 2008 ▶); program(s) used to refine structure: *SHELXTL*; molecular graphics: *SHELXTL*; software used to prepare material for publication: *SHELXTL* and *PLATON* (Spek, 2009 ▶).

## Supplementary Material

Crystal structure: contains datablocks global, I. DOI: 10.1107/S1600536810052621/ng5089sup1.cif

Structure factors: contains datablocks I. DOI: 10.1107/S1600536810052621/ng5089Isup2.hkl

Additional supplementary materials:  crystallographic information; 3D view; checkCIF report

## Figures and Tables

**Table 1:** Hydrogen-bond geometry (Å, °) *Cg*3 is the centroid of the C1–C6 ring.

*D*—H⋯*A*	*D*—H	H⋯*A*	*D*⋯*A*	*D*—H⋯*A*
C11—H11*B*⋯*Cg*3^i^	0.97	2.70	3.544 (2)	145

Symmetry code: (i) 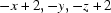.

**Fig. 1.**
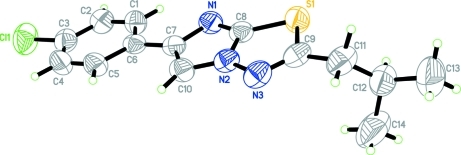
The asymmetric unit of the title compound, showing 50% probability displacement ellipsoids and the atom-numbering scheme.

**Fig. 2.**
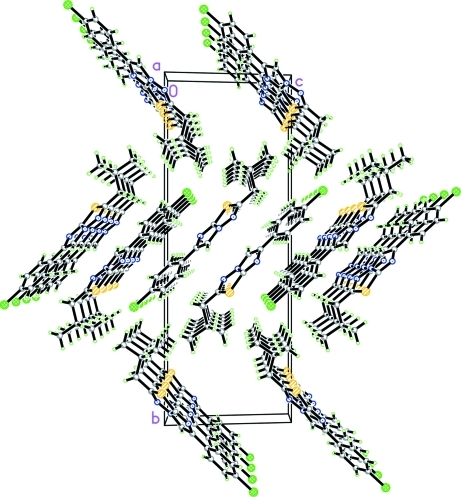
The crystal packing of the title compound (I).

## supplementary crystallographic information

### Comment

Imidazo [2,1-*b*]-1,3,4-thiadiazole derivatives are found to be biologically active compounds possessing anticancer (Terzioglu, 2003), antitubercular (Kolavi *et al.*, 2006), antibacterial (Gadad *et al.*, 2000), antifungal (Andotra *et al.*, 1997), anticonvulsant, analgesic (Khazi *et al.*, 1996), anti-inflammatory (Andreani *et al.*, 1982), diuretic (Andreani *et al.*, 1991) and herbicidal activities (Andreani *et al.*, 1991). Moreover 1,3,4-thiadiazoles have many interesting biological activities, for example, 2-amino-5-(trifluoromethylphenyl alkyl)-1,3,4 thidadiazoles are used in the treatment of insomnia and anxiety (Eberle & Robert, 1977).

The asymmetric unit of the title compound is shown in Fig. 1. The imidazo[2,1-*b*] [1,3,4]thiadiazole (S1/N1–N3/C7–C10) ring is essentially planar, with a maximum deviation of 0.006 (2) Å for atom N3. The dihedral angle between the imidazo[2,1-*b*][1,3,4]thiadiazole (S1/N1–N3/C7–C10) ring and the chlorophenyl ring (C1–C6) is 5.07 (8)°.

In the crystal structure (Fig. 2), there are no classical hydrogen bonds but stabilization is provided by weak π–π interactions between the imidazole rings (N1–N2/C7–C8/C10) [centroid-to-centroid (2-x, -y, 2-z) distance = 3.5697 (11) Å].  Furthermore, the crystal structure is stabilized by C—H···π interactions (Table 1), involving the (C1–C6)(centroid Cg3) ring.

### Experimental

5-Isobutyl-1,3,4-thiadiazole-2-amine( 1 equivalent) and 4-chlorophenacyl bromide (1 equivalent) are refluxed with ethanol for 4 hrs.  The solvent was then distilled off and the reaction mass was poured onto crushed ice. The resulting solid, 6-(4-chlorophenyl)-2-isobutylimidazo[2,1-*b*] [1,3,4]thiadiazole, that separated out was filtered and dried.  The compound was recrystallized using ethanol and DMF mixture. M.pt. 121–126 °C.

### Refinement

All the H atoms were positioned geometrically [C–H = 0.93–0.97 Å] and were refined using a riding model, with *U*_iso_(H) = 1.2 or 1.5 *U*_eq_(C).

### Crystal data

**Table:**

C_14_H_14_ClN_3_S	*F*(000) = 608
*M**_r_* = 291.79	*D*_x_ = 1.332 Mg m^−^^3^
Monoclinic, *P*2_1_/*n*	Mo *K*α radiation, λ = 0.71073 Å
Hall symbol: -P 2yn	Cell parameters from 5357 reflections
*a* = 5.7552 (1) Å	θ = 2.3–28.5°
*b* = 26.4052 (5) Å	µ = 0.40 mm^−^^1^
*c* = 9.7662 (2) Å	*T* = 296 K
β = 101.388 (1)°	Block, colourless
*V* = 1454.92 (5) Å^3^	0.47 × 0.31 × 0.28 mm
*Z* = 4	

### Data collection

**Table:**

Bruker SMART APEXII CCD area-detector diffractometer	3353 independent reflections
Radiation source: fine-focus sealed tube	2646 reflections with *I* > 2σ(*I*)
graphite	*R*_int_ = 0.023
φ and ω scans	θ_max_ = 27.5°, θ_min_ = 2.3°
Absorption correction: multi-scan (*SADABS*; Bruker, 2009)	*h* = −6→7
*T*_min_ = 0.835, *T*_max_ = 0.897	*k* = −23→34
12320 measured reflections	*l* = −12→10

### Refinement

**Table:**

Refinement on *F*^2^	Primary atom site location: structure-invariant direct methods
Least-squares matrix: full	Secondary atom site location: difference Fourier map
*R*[*F*^2^ > 2σ(*F*^2^)] = 0.050	Hydrogen site location: inferred from neighbouring sites
*wR*(*F*^2^) = 0.135	H-atom parameters constrained
*S* = 1.05	*w* = 1/[σ^2^(*F*_o_^2^) + (0.0605*P*)^2^ + 0.4615*P*] where *P* = (*F*_o_^2^ + 2*F*_c_^2^)/3
3353 reflections	(Δ/σ)_max_ < 0.001
172 parameters	Δρ_max_ = 0.23 e Å^−^^3^
0 restraints	Δρ_min_ = −0.32 e Å^−^^3^

### Special details

**Table:**

Geometry. All esds (except the esd in the dihedral angle between two l.s. planes) are estimated using the full covariance matrix. The cell esds are taken into account individually in the estimation of esds in distances, angles and torsion angles; correlations between esds in cell parameters are only used when they are defined by crystal symmetry. An approximate (isotropic) treatment of cell esds is used for estimating esds involving l.s. planes.
Refinement. Refinement of F^2^ against ALL reflections. The weighted R-factor wR and goodness of fit S are based on F^2^, conventional R-factors R are based on F, with F set to zero for negative F^2^. The threshold expression of F^2^ > 2sigma(F^2^) is used only for calculating R-factors(gt) etc. and is not relevant to the choice of reflections for refinement. R-factors based on F^2^ are statistically about twice as large as those based on F, and R- factors based on ALL data will be even larger.

### Fractional atomic coordinates and isotropic or equivalent isotropic displacement parameters (Å^2^)

**Table:**

	*x*	*y*	*z*	*U*_iso_*/*U*_eq_	
Cl1	0.79047 (16)	−0.15309 (3)	0.31411 (7)	0.0936 (3)	
S1	0.87829 (9)	0.11339 (2)	0.99277 (7)	0.0661 (2)	
N1	0.8370 (3)	0.03334 (6)	0.79472 (18)	0.0536 (4)	
N2	1.1813 (3)	0.05331 (6)	0.93125 (17)	0.0517 (4)	
N3	1.3144 (3)	0.08204 (7)	1.03504 (19)	0.0609 (5)	
C1	0.7404 (4)	−0.04026 (8)	0.5763 (2)	0.0544 (5)	
H1A	0.6230	−0.0182	0.5931	0.065*	
C2	0.6859 (4)	−0.07545 (8)	0.4702 (2)	0.0610 (5)	
H2A	0.5347	−0.0767	0.4147	0.073*	
C3	0.8587 (4)	−0.10854 (8)	0.4479 (2)	0.0617 (5)	
C4	1.0837 (4)	−0.10677 (9)	0.5281 (3)	0.0686 (6)	
H4A	1.1991	−0.1294	0.5116	0.082*	
C5	1.1370 (4)	−0.07123 (8)	0.6331 (2)	0.0613 (5)	
H5A	1.2889	−0.0701	0.6877	0.074*	
C6	0.9657 (3)	−0.03694 (7)	0.65853 (19)	0.0471 (4)	
C7	1.0177 (3)	0.00199 (7)	0.76767 (19)	0.0468 (4)	
C8	0.9462 (3)	0.06335 (7)	0.8937 (2)	0.0507 (4)	
C9	1.1782 (4)	0.11490 (8)	1.0758 (2)	0.0578 (5)	
C10	1.2301 (3)	0.01388 (8)	0.8507 (2)	0.0544 (5)	
H10A	1.3760	−0.0015	0.8522	0.065*	
C11	1.2613 (5)	0.15173 (9)	1.1910 (2)	0.0731 (6)	
H11A	1.4291	0.1461	1.2261	0.088*	
H11B	1.1795	0.1445	1.2666	0.088*	
C12	1.2254 (5)	0.20690 (10)	1.1527 (3)	0.0813 (7)	
H12A	1.0588	0.2118	1.1082	0.098*	
C13	1.2777 (9)	0.23946 (14)	1.2842 (4)	0.1384 (16)	
H13A	1.1748	0.2297	1.3459	0.208*	
H13B	1.2516	0.2745	1.2591	0.208*	
H13C	1.4397	0.2347	1.3303	0.208*	
C14	1.3764 (8)	0.22228 (13)	1.0505 (4)	0.1297 (15)	
H14A	1.3400	0.2011	0.9691	0.195*	
H14B	1.5407	0.2185	1.0930	0.195*	
H14C	1.3448	0.2570	1.0240	0.195*	

### Atomic displacement parameters (Å^2^)

**Table:**

	*U*^11^	*U*^22^	*U*^33^	*U*^12^	*U*^13^	*U*^23^
Cl1	0.1320 (7)	0.0746 (4)	0.0756 (4)	0.0014 (4)	0.0239 (4)	−0.0144 (3)
S1	0.0529 (3)	0.0606 (3)	0.0842 (4)	0.0008 (2)	0.0124 (3)	−0.0096 (3)
N1	0.0371 (8)	0.0555 (10)	0.0668 (10)	0.0018 (7)	0.0068 (7)	0.0014 (8)
N2	0.0405 (8)	0.0511 (9)	0.0604 (9)	−0.0010 (7)	0.0024 (7)	0.0085 (7)
N3	0.0512 (9)	0.0583 (10)	0.0671 (11)	−0.0053 (8)	−0.0031 (8)	0.0042 (8)
C1	0.0496 (10)	0.0545 (11)	0.0571 (11)	0.0090 (8)	0.0055 (8)	0.0064 (9)
C2	0.0614 (12)	0.0627 (13)	0.0556 (11)	0.0045 (10)	0.0034 (9)	0.0072 (10)
C3	0.0819 (15)	0.0540 (12)	0.0523 (11)	0.0005 (10)	0.0207 (10)	0.0066 (9)
C4	0.0682 (14)	0.0641 (14)	0.0796 (15)	0.0138 (11)	0.0292 (12)	0.0028 (11)
C5	0.0460 (10)	0.0638 (13)	0.0753 (14)	0.0076 (9)	0.0149 (10)	0.0042 (11)
C6	0.0441 (9)	0.0481 (10)	0.0503 (10)	0.0030 (7)	0.0122 (7)	0.0138 (8)
C7	0.0386 (9)	0.0475 (10)	0.0541 (10)	0.0027 (7)	0.0088 (7)	0.0129 (8)
C8	0.0392 (9)	0.0492 (10)	0.0637 (11)	−0.0002 (8)	0.0103 (8)	0.0074 (9)
C9	0.0599 (11)	0.0516 (11)	0.0594 (12)	−0.0095 (9)	0.0055 (9)	0.0108 (9)
C10	0.0398 (9)	0.0570 (11)	0.0650 (12)	0.0078 (8)	0.0069 (8)	0.0076 (9)
C11	0.0872 (17)	0.0662 (14)	0.0625 (13)	−0.0168 (12)	0.0068 (12)	0.0047 (11)
C12	0.0844 (17)	0.0661 (15)	0.0928 (18)	−0.0077 (13)	0.0157 (14)	−0.0147 (13)
C13	0.193 (4)	0.103 (3)	0.133 (3)	−0.032 (3)	0.066 (3)	−0.052 (2)
C14	0.208 (4)	0.088 (2)	0.104 (2)	−0.059 (2)	0.055 (3)	−0.0064 (18)

### Geometric parameters (Å, °)

**Table:**

Cl1—C3	1.744 (2)	C5—H5A	0.9300
S1—C8	1.727 (2)	C6—C7	1.468 (3)
S1—C9	1.757 (2)	C7—C10	1.363 (3)
N1—C8	1.311 (3)	C9—C11	1.492 (3)
N1—C7	1.395 (2)	C10—H10A	0.9300
N2—C8	1.356 (2)	C11—C12	1.508 (3)
N2—C10	1.367 (3)	C11—H11A	0.9700
N2—N3	1.372 (2)	C11—H11B	0.9700
N3—C9	1.284 (3)	C12—C14	1.503 (4)
C1—C2	1.381 (3)	C12—C13	1.525 (4)
C1—C6	1.387 (3)	C12—H12A	0.9800
C1—H1A	0.9300	C13—H13A	0.9600
C2—C3	1.373 (3)	C13—H13B	0.9600
C2—H2A	0.9300	C13—H13C	0.9600
C3—C4	1.376 (3)	C14—H14A	0.9600
C4—C5	1.379 (3)	C14—H14B	0.9600
C4—H4A	0.9300	C14—H14C	0.9600
C5—C6	1.397 (3)		
			
C8—S1—C9	88.02 (10)	N3—C9—C11	123.3 (2)
C8—N1—C7	103.40 (15)	N3—C9—S1	116.48 (16)
C8—N2—C10	107.48 (16)	C11—C9—S1	120.14 (18)
C8—N2—N3	118.26 (17)	C7—C10—N2	104.82 (16)
C10—N2—N3	134.26 (16)	C7—C10—H10A	127.6
C9—N3—N2	108.46 (16)	N2—C10—H10A	127.6
C2—C1—C6	121.66 (19)	C9—C11—C12	115.8 (2)
C2—C1—H1A	119.2	C9—C11—H11A	108.3
C6—C1—H1A	119.2	C12—C11—H11A	108.3
C3—C2—C1	118.9 (2)	C9—C11—H11B	108.3
C3—C2—H2A	120.6	C12—C11—H11B	108.3
C1—C2—H2A	120.6	H11A—C11—H11B	107.4
C2—C3—C4	121.2 (2)	C14—C12—C11	110.9 (3)
C2—C3—Cl1	118.99 (18)	C14—C12—C13	111.3 (3)
C4—C3—Cl1	119.80 (18)	C11—C12—C13	110.0 (3)
C3—C4—C5	119.5 (2)	C14—C12—H12A	108.2
C3—C4—H4A	120.2	C11—C12—H12A	108.2
C5—C4—H4A	120.2	C13—C12—H12A	108.2
C4—C5—C6	120.8 (2)	C12—C13—H13A	109.5
C4—C5—H5A	119.6	C12—C13—H13B	109.5
C6—C5—H5A	119.6	H13A—C13—H13B	109.5
C1—C6—C5	117.93 (19)	C12—C13—H13C	109.5
C1—C6—C7	119.84 (17)	H13A—C13—H13C	109.5
C5—C6—C7	122.23 (17)	H13B—C13—H13C	109.5
C10—C7—N1	111.42 (17)	C12—C14—H14A	109.5
C10—C7—C6	128.53 (17)	C12—C14—H14B	109.5
N1—C7—C6	120.03 (15)	H14A—C14—H14B	109.5
N1—C8—N2	112.88 (17)	C12—C14—H14C	109.5
N1—C8—S1	138.34 (15)	H14A—C14—H14C	109.5
N2—C8—S1	108.78 (14)	H14B—C14—H14C	109.5
			
C8—N2—N3—C9	−0.8 (2)	C7—N1—C8—S1	179.56 (18)
C10—N2—N3—C9	179.7 (2)	C10—N2—C8—N1	0.2 (2)
C6—C1—C2—C3	1.3 (3)	N3—N2—C8—N1	−179.44 (16)
C1—C2—C3—C4	−0.7 (3)	C10—N2—C8—S1	−179.60 (13)
C1—C2—C3—Cl1	179.91 (16)	N3—N2—C8—S1	0.8 (2)
C2—C3—C4—C5	0.2 (3)	C9—S1—C8—N1	179.9 (2)
Cl1—C3—C4—C5	179.57 (17)	C9—S1—C8—N2	−0.42 (14)
C3—C4—C5—C6	−0.3 (3)	N2—N3—C9—C11	178.09 (18)
C2—C1—C6—C5	−1.4 (3)	N2—N3—C9—S1	0.4 (2)
C2—C1—C6—C7	178.29 (18)	C8—S1—C9—N3	−0.01 (17)
C4—C5—C6—C1	0.9 (3)	C8—S1—C9—C11	−177.76 (18)
C4—C5—C6—C7	−178.82 (19)	N1—C7—C10—N2	0.1 (2)
C8—N1—C7—C10	0.0 (2)	C6—C7—C10—N2	178.64 (17)
C8—N1—C7—C6	−178.69 (16)	C8—N2—C10—C7	−0.1 (2)
C1—C6—C7—C10	−174.21 (19)	N3—N2—C10—C7	179.36 (19)
C5—C6—C7—C10	5.5 (3)	N3—C9—C11—C12	122.0 (3)
C1—C6—C7—N1	4.2 (3)	S1—C9—C11—C12	−60.4 (3)
C5—C6—C7—N1	−176.07 (17)	C9—C11—C12—C14	−66.3 (3)
C7—N1—C8—N2	−0.1 (2)	C9—C11—C12—C13	170.2 (3)

### Hydrogen-bond geometry (Å, °)

**Table:**

Cg3 is the centroid of the C1–C6 ring.

**Table:**

*D*—H···*A*	*D*—H	H···*A*	*D*···*A*	*D*—H···*A*
C11—H11B···Cg3^i^	0.97	2.70	3.544 (2)	145

Symmetry codes: (i) −*x*+2, −*y*, −*z*+2.
